# Local Health Department Epidemiologic Capacity: A Stratified Cross-Sectional Assessment Describing the Quantity, Education, Training, and Perceived Competencies of Epidemiologic Staff

**DOI:** 10.3389/fpubh.2013.00064

**Published:** 2013-12-02

**Authors:** Kaitlin A. O’Keefe, Shira C. Shafir, Kimberley I. Shoaf

**Affiliations:** ^1^Center for Public Health and Disasters, Fielding School of Public Health, University of California Los Angeles, Los Angeles, CA, USA; ^2^Department of Epidemiology, Fielding School of Public Health, University of California Los Angeles, Los Angeles, CA, USA; ^3^Department of Health Sciences, California State University Northridge, Northridge, CA, USA; ^4^Department of Community Health Sciences, Fielding School of Public Health, University of California Los Angeles, Los Angeles, CA, USA

**Keywords:** epidemiologist, local health department, capacity, workforce, epidemiologic training

## Abstract

**Introduction**: Local health departments (LHDs) must have sufficient numbers of staff functioning in an epidemiologic role with proper education, training, and skills to protect the health of communities they serve. This pilot study was designed to describe the composition, training, and competency level of LHD staff and examine the hypothesis that potential disparities exist between LHDs serving different sized populations.

**Materials and Methods**: Cross-sectional surveys were conducted with directors and epidemiologic staff from a sample of 100 LHDs serving jurisdictions of varied sizes. Questionnaires included inquiries regarding staff composition, education, training, and measures of competency modeled on previously conducted studies by the Council of State and Territorial Epidemiologists. Number of epidemiologic staff, academic degree distribution, epidemiologic training, and both director and staff confidence in task competencies were calculated for each LHD size strata.

**Results**: Disparities in measurements were observed in LHDs serving different sized populations. LHDs serving small populations reported a smaller average number of epidemiologic staff than those serving larger jurisdictions. As size of population served increased, percentages of staff and directors holding bachelors’ and masters’ degrees increased, while those holding RN degrees decreased. A higher degree of perceived competency of staff in most task categories was reported in LHDs serving larger populations.

**Discussion**: LHDs serving smaller populations reported fewer epidemiologic staff, therefore might benefit from additional resources. Differences observed in staff education, training, and competencies suggest that enhanced epidemiologic training might be particularly needed in LHDs serving smaller populations. Results can be used as a baseline for future research aimed at identifying areas where training and personnel resources might be particularly needed to increase the capabilities of LHDs.

## Introduction

With the ever present threat of bioterrorism, emerging and reemerging infectious diseases, and a steady increase in the incidence of many chronic diseases, health departments must be fully prepared for a multitude of tasks in order to protect and promote the health of the communities for which they are responsible. Many of the duties essential to a fully functioning health department include epidemiologic tasks such as disease surveillance, study design, data collection and analysis, and the design of disease control measures (1). An adequate number of educated and trained personnel is required to successfully complete these tasks and ensure that a health department is able to sufficiently fulfill its necessary responsibilities within a community. Although such duties within local health departments (LHDs) are often performed by employed epidemiologists, oftentimes the size of health departments will necessitate the assignment of many of these duties to staff members outside the designation of epidemiologist, such as public health nurses (1). Due to the variety of responsibilities entrusted to public health agencies, particularly with regard to emergency preparedness needs, it has been suggested that all public health workers should have certain levels of knowledge and skill, or competency, in performing epidemiologic tasks (2, 3). A heightened level of task competency of all department staff members functioning in epidemiologic roles could increase the total epidemiologic capacity of LHDs in the United States, enhancing their ability to efficiently detect, prevent, and control public health-related issues (4). A first step in ensuring that these needs are met must include an assessment of the quantity, level of education and training of staff fulfilling these roles within health departments, as well as the levels of staff competency in performing epidemiologic tasks.

Several previous assessments have attempted to quantify the number and type of staff employed in LHDs throughout the country, including studies conducted by the National Association of County and City Health Officials (NACCHO) and the Council of State and Territorial Epidemiologists (CSTE) (5, 6). Although each of these reports provides valuable information describing the workforce in LHDs, highlighting departments and program areas with potential staff shortages, neither report includes specific details on the training and education level of employed epidemiologists or those performing an epidemiologic role within the health department. The CSTE, an organization representing public health epidemiologists in US states and territories, has also conducted periodic assessments of the epidemiologic capacity of state and territory health departments in the United States since 2001, which include measures of epidemiologic staff competency in a variety of defined tasks (7). The assessments conducted in 2001, 2004, 2006, and 2009 using the CSTE Epidemiology Capacity Assessment (ECA) tool, yielded significant findings, including notably suboptimal levels of epidemiologic capacity of state and territorial-level health departments (7–10). While this ongoing project has proven to be a valuable tool in the assessment and guidance of public health systems nationwide at a state level, similar evaluations of health departments on a local level have not been conducted, representing a critical gap in knowledge. As LHDs are often the first to respond in any number of health situations, it is important to understand the capacity of each individual LHD.

Other previous studies have been conducted detailing descriptions of those working in epidemiologic roles within a health department, several of which additionally describe various measures of staff competency (11–23). Although these earlier studies provide important insight into the capacity of health departments in the United States, most have been conducted at a state level health agency, within specific programs of a local health agency, or focusing on total agency or program capacity.

To our knowledge, no studies exist which attempt to comprehensively evaluate the epidemiologic capacity of LHDs throughout the United States, using a composite of variables designed to estimate the number of epidemiologic staff, level of education, and epidemiologic training of those employed and level of staff competency in various epidemiologic tasks.

Based on the models used by the CSTE and NACCHO, this pilot study was designed to provide an initial description of the composition, training, and competency level of staff members performing epidemiologic functions within a small sample of LHDs. This study further examines the hypothesis that potential disparities in staff size, education, and confidence levels in epidemiologic competencies may exist between health departments serving different sized populations. This pilot study was intended to establish a baseline description of measures of the epidemiologic capacity of LHDs on a small scale. An additional goal of this pilot was to develop a potential model to be used for further research on a larger scale. The baseline description from results can be used to provide evidence of the need for future assessments of this nature to explore components of LHD epidemiologic capacity.

## Materials and Methods

To conduct the assessment and test the hypothesis described, two questionnaires were developed and distributed to a sample of city and county health departments throughout the country. As this was designed as a pilot study to examine epidemiologic capacity and size disparities using the tools described below, a small sample consisting of 100 LHDs was drawn. IRB approval was obtained from the South General IRB at the University of California Los Angeles. Informed consent was obtained for all participants.

### Sampling

The sample of health departments used for this study was created by referencing the list of LHDs compiled by NACCHO. At the time of study initiation, the most recent and comprehensive list of LHDs available consisted of data from the NACCHO 2008 National Profile of LHDs (24). A LHD was defined as “an administrative or service unit of local or state government, concerned with health and carrying some responsibility for the health of a jurisdiction smaller than the state” (24). The 2008 Profile included information from 2,332 LHDs nationwide, an 83% response rate from the 2,794 LHDs in the 2008 Profile study population (24).

The list of the 2,332 LHDs which submitted information for the 2008 Profile was first stratified based on the reported size of population served. Categories of population sizes used by NACCHO were condensed into small, medium, and large categories for the purposes of this study due to sample size constraints of the intended small scale of the pilot. A small LHD was considered as one that serves a population of less than 25,000 people, a medium LHD as one that serves a population of 25,000–250,000 people, and a large LHD as one that serves a population greater than 250,000 people.

As this study was designed as small scale pilot, the authors decided on an initial sample size of 100 LHDs. In the 2008 NACCHO Profile, the number of LHD in the combined categories approximated 39.1, 50.6, and 10.3% for small, medium, and large health departments respectively (24). To ensure enough large health departments were included in the sample, authors decided to increase the proportion of large health departments included to 20% of the initial sample. A stratified random sample was drawn of 40 small, 40 medium, and 20 large LHDs using SAS version 9.2 (SAS Institute, Cary, NC, USA). The list of 100 LHDs was reviewed, using departmental website information and contact via phone, for eligibility in our study based on criteria defined for study purposes to include only “full-service” health departments. This was primarily done to minimize the chance an unrepresentative health department that was not intended to fulfill the same needs as a “full-service” health department was included. A LHD met the inclusion criteria if they self-reportedly performed at least 6 of the 10 “Essential Public Health Services” and provided at least four separate public health services to the community including, but not limited to, communicable disease control, maternal and child health services, environmental control, immunizations, vital statistics, and health education (25). If a selected LHD did not meet the inclusion criteria, they were excluded from the sample and another LHD from the same stratum was randomly selected and evaluated.

### Questionnaire development

Two questionnaires were developed, one intended for the director of each LHD and one intended for epidemiologic staff at each LHD. Epidemiologic staff members were identified by the directors of the LHD as individuals performing epidemiologic functions within the health department, using the CSTE criteria for an epidemiologist (8). The CSTE assessment identified an epidemiologist as any person who performed functions consistent with the definition of an epidemiologist provided in *A Dictionary of Epidemiology* as “an investigator who studies the occurrence of disease or other health-related conditions or events in defined populations. The control of disease in populations is often also considered to be a task for the epidemiologist” (8, 26). This was done to ensure that health departments without a designated position of “epidemiologist,” but with staff who performed epidemiologic functions within their position, would be represented to best assess the epidemiologic capabilities of a department.

The design of each questionnaire was largely based on the models used by the CSTE in the periodic ECA tools and by NACCHO in the National Profile (5, 8). Competency questions were modeled after competency questions from the 2009 CSTE ECA tool (8). The questionnaire designed for the LHD directors contained items regarding the total number of epidemiologic staff at their current LHD. Previous education and training received by directors, both in general academic degrees obtained and highest level of training received in epidemiology, were also assessed. Directors were asked to indicate whether they had received one or more of the following degrees: High School Diploma/GED (General Educational Development test); Associates; RN (Registered Nursing) or any other nursing; any bachelors degree including Bachelor of Arts (BA), Bachelor of Science (BS), Bachelor of Science in Nursing (BSN), or other; any masters degree including Master of Public Health (MPH), Master of Science in Public Health (MSPH) or other; any doctoral degree including Doctor of Philosophy (PhD), Doctor of Public Health (DrPH), or other doctoral; Doctor of Veterinary Medicine (DVM); Doctor of Dental Surgery (DDS); Doctor of Medicine (MD) or Doctor of Osteopathic Medicine (DO). Directors were also asked about the highest level of training obtained specifically in epidemiology, with the following possible options: no formal training in epidemiology; received continuing education training in epidemiology at your current and/or a previous job; completed some coursework in epidemiology; completed formal training program in epidemiology [e.g., Epidemic Intelligence Service (EIS)]; BA, BS, other bachelor degree in epidemiology; MPH, MSPH, other master degree in epidemiology; professional background (e.g., MD) with a dual degree in epidemiology; PhD, DrPH, other doctoral degree in epidemiology. The questionnaire also contained measures of competencies to evaluate the directors’ level of confidence in the abilities of their self-reported epidemiologic staff in performing designated epidemiologic functions. Questions on confidence in competencies were designed on a Likert scale, ranging from “Not at all Confident (1)” to “Completely Confident (5),” with an available option of “Don’t Know/Unsure.”

The questionnaire intended for distribution to epidemiologic staff contained questions similarly designed to the directors’ questionnaires regarding the amount and type of education and training received both before and during their time employed by their current LHD. Additional questions were included that were designed to measure individuals’ assessments of confidence in their own abilities to perform designated epidemiologic functions, based on the same questions and measurement scales regarding confidence in competencies used in the directors’ questionnaire.

### Distribution

The name and contact email of the director at each LHD was obtained through departmental website review and/or contact via phone. In certain instances, a general departmental email address or email address for an alternate contact was provided instead. Invitations for study participation containing a link to access the online questionnaire were sent to the director at each LHD via the email address provided. This individual was asked to first complete the corresponding director questionnaire, and then forward the link for the staff questionnaire to an employe they identified as performing epidemiologic functions within their health department for completion. A link to descriptions of defined epidemiologic functions for the purposes of this study was provided for clarity. This was done to ensure individuals who did not have the title of “epidemiologist” but who regularly performed epidemiologic functions within their health department were appropriately represented.

If a response was not received from a health department by the initial deadline of 1 month after distribution, follow-up telephone calls were made and the questionnaire was completed through phone interview once participants reviewed the study information and consented to participate. Responses from both directors and epidemiologic staff were coded and remained anonymous.

### Analysis

Once data collection was complete, stratified analyses of the results by size of population served by each health department were performed using SAS 9.2 (SAS Institute, Cary, NC, USA). The mean number of epidemiologic staff was calculated and the distribution of academic degrees and highest level of training in epidemiology obtained were reported as percentages in each of the strata for both directors and staff members. Questions on confidence in competencies on the Likert scale were collapsed into broader categories of variables for ease of interpretability. The mean score of responses on confidence in categories of competency measures included in the questionnaire were calculated for both director and staff responses for each of the three strata. As responses from directors and staff were collected from different health departments in many cases and were coded to ensure anonymity, no attempt was made to calculate measures of agreement between director and staff perceptions of competency. Responses of “Don’t Know/Unsure” were excluded from mean calculations within each measure. As this was a pilot study with a low initial sample size designed primarily for a descriptive analysis of observations, measures to assess the statistical significance of observed differences between health departments of different sizes were not calculated.

## Results

Response rates varied for the director and epidemiologic staff questionnaire in each of the strata. A total of 45 responses were collected from directors of LHDs. There were 19 out of 40 responses collected from small jurisdictions, 17 out of 40 from medium jurisdictions, and 9 out of 20 from large jurisdictions representing response rates of 47.5, 42.5, and 45% respectively. There were 27 responses collected from epidemiologic staff members: 6 out of 40 from small jurisdictions, 9 out of 40 from medium jurisdictions, and 12 out of 20 from large jurisdictions, representing response rates of 15, 22.5, and 60% respectively.

Results of the directors’ questionnaire responses regarding the quantity of staff performing epidemiologic functions within their respective LHDs are presented in Table 1.

**Table 1:** **Average number of individuals performing epidemiologic functions within local health departments (LHDs.)**.

Small (*N* = 19)	Medium (*N* = 17)	Large (*N* = 9)
2.4	5.8	10.9

Small LHD = < 25,000 population, medium LHD = 25,000–250,000 population, large LHD > 250,000 population.

Supporting the initial study hypothesis, smaller jurisdictions tended to have a smaller number of staff members classified as performing epidemiologic functions within the department. The average number of staff members performing epidemiologic functions within health departments serving small jurisdictions (2.4 epidemiologic employees) was less than half that in departments serving medium jurisdictions (5.8 epidemiologic employees) and close to a fifth of the average number in departments serving large jurisdictions (10.9 epidemiologic employees).

The academic degree distribution for both directors and epidemiologic staff members in LHDs are presented in Table 2.

**Table 2:** **Distribution of academic degrees obtained by directors and epidemiologic staff at local health departments (LHDs)**.

Degrees	Director			Epidemiologic staff		
	Small (*N* = 19)	Medium (*N* = 17)	Large (*N* = 9)	Small (*N* = 6)	Medium (*N* = 9)	Large (*N* = 12)
	*N* (%)			*N* (%)		
Associate	6 (31.6)	4 (23.5)	0 (0)	3 (50)	2 (22.2)	0 (0)
RN, any other nursing	10 (52.6)	7 (41.2)	2 (22.2)	6 (100)	5 (55.6)	0 (0)
BA, BS, BSN, other bachelor	13 (68.4)	14 (82.4)	9 (100)	3 (50)	8 (88.9)	12 (100)
MPH, MSPH, other master	4 (21.1)	8 (47.1)	8 (88.9)	0 (0)	2 (22.2)	12 (100)
PhD, DrPH, other doctoral	0 (0)	0 (0)	0 (0)	0 (0)	0 (0)	0 (0)
MD, DO	0 (0)	1 (5.9)	2 (22.2)	0 (0)	0 (0)	1 (8.3)

Small LHD = < 25,000 population, medium LHD = 25,000–250,000 population, large LHD > 250,000 population.

Noticeable differences in the degree distribution of both directors and epidemiologic staff members were seen in the results and, as hypothesized, among departments serving areas of different sizes. As the size of the jurisdictional area increased, the percentage of both directors and staff holding bachelors and masters degrees increased, while the percentage of both directors and staff holding RN degrees decreased. No director or staff member interviewed held a PhD or other academic doctoral degree, and only three directors and one staff member held an MD or DO in the combined sample.

Responses regarding highest level of training obtained in epidemiology among both directors and staff members are presented in Table 3.

**Table 3:** **Highest level of training obtained in epidemiology of directors and epidemiologic staff employed at local health departments, by percentage of group that have obtained training**.

Level of training	Director			Epidemiologic staff		
	Small (*N* = 19)	Medium (*N* = 17)	Large (*N* = 9)	Small (*N* = 6)	Medium (*N* = 9)	Large (*N* = 12)
No formal training in epidemiology	26.3	17.7	0	33.3	33.3	0
Received continuing education training in epidemiology at your current and/or a previous job	31.6	23.5	11.1	33.3	33.3	8.3
Completed some coursework in epidemiology	36.8	52.9	55.6	33.3	11.1	16.7
Completed formal training program in epidemiology (e.g., EIS)	5.3	5.9	22.2	0	0	0
BA, BS, other bachelors degree in epidemiology	0	0	0	0	0	0
MPH, MSPH, other masters degree in epidemiology	0	0	11.1	0	22.2	66.7
Professional background (e.g., MD) with a dual degree in epidemiology	0	0	0	0	0	8.3
PhD, DrPH, other doctoral degree in epidemiology	0	0	0	0	0	0

Small LHD = < 25,000 population, medium LHD = 25,000–250,000 population, large LHD > 250,000 population.

A third of staff members in departments serving small and medium jurisdictions reported having received “no formal training in epidemiology,” while no epidemiologic staff member reported this response at health departments serving large jurisdictions. More than a quarter and a sixth of directors in departments serving small and medium health jurisdictions, respectively, reported having received “no formal training in epidemiology,” while no director reported this in the large stratum. The majority of epidemiologic staff members (66.7%) in large health departments reported having a masters degree in epidemiology, while the percentage was much smaller in small and medium health departments (0 and 22.2% respectively). Very few directors and no staff members reported having “completed a formal training program in epidemiology (e.g., EIS).” One third or less of staff members and directors reported that the highest level of epidemiologic training that they had received was “continuing education training in epidemiology at your current and/or a previous job.”

The mean score of responses from variables on confidence collapsed into broader categories of epidemiologic competencies are presented in Table 4. As hypothesized, differences were observed in confidence levels between differing strata of health departments. Both directors and staff members at LHDs serving large populations had the highest average confidence ratings across all categories of epidemiologic competencies when compared to LHDs serving small and medium populations. LHD directors and epidemiologic staff serving large jurisdictions consistently rated their confidence in competency level higher than that of directors and staff from LHDs serving smaller jurisdictions. Lowest reported confidence levels varied between small and medium strata for both LHD directors and epidemiologic staff within different competency categories.

**Table 4:** **Average scores in confidence of directors and epidemiologic staff members of local health departments in categories of collapsed epidemiologic competencies**.

Epidemiologic competencies categories	Director			Epidemiologic staff		
	Small (*N* = 19)	Medium (*N* = 17)	Large (*N* = 9)	Small (*N* = 6)	Medium (*N* = 9)	Large (*N* = 12)
Essential services of public health	3.37	3.09	3.97	3.13	3.25	4.15
Understanding epidemiologic functions and public health purpose and practices	3.39	3.35	3.79	3.86	3.83	4.53
Identifying public health issues	3.71	3.83	4.37	3.69	3.91	4.54
Development and design of public health programs and projects	3.01	3.13	4.17	3.05	3.60	4.29
Public health actions	3.44	3.53	4.13	3.56	3.41	4.24
Collection of data	2.81	2.84	4.36	2.63	3.00	4.77
Analysis of data	2.56	2.31	4.11	2.17	3.04	4.47
Interpreting results from analysis	2.95	3.03	4.18	2.86	3.51	4.54
Conveying/communicating results of data	3.49	3.22	4.11	3.14	2.73	4.58
Evaluation/assessment of systems	3.70	3.29	4.07	3.33	3.63	4.22
Following ethical guidelines	3.30	3.27	4.53	3.67	3.67	4.63
Able to work together with the community	3.28	3.35	4.15	3.67	3.33	4.22
Knowledge of agency policies and practical involvement in agency mission	4.10	3.78	4.38	Not asked	Not asked	Not asked

Averages are based on a 1–5 scale of level of confidence; 1 = not at all confident, 5 = completely confident.

Small LHD = < 25,000 population, medium LHD = 25,000–250,000 population, large LHD > 250,000 population.

Looking within individual strata, directors from LHDs serving large populations had the highest average level of confidence in their epidemiologic staff in the category of “Following Ethical Concerns and Guidelines” (4.53), while staff members from the same LHD stratum had the highest level of confidence in the “Collection of Data” category (4.77). Both directors and staff from LHDs serving medium populations had the highest average level of confidence in their epidemiologic staff in the category of “Identifying Public Health Issues” (3.83 and 3.91 for directors and staff respectively). Directors from LHDs serving small populations had the highest average level of confidence in their staff in the category of “Knowledge of Agency Policies and Practical Involvement in Agency Mission” (4.10), while staff members in the same LHD stratum had the highest level of confidence in “Understanding Epidemiologic Functions and Public Health Purpose and Practices” (3.86).

## Discussion

The results from this study support the initial study hypothesis that important potential differences in the quantity, education, training, and competency level of epidemiologic staff may exist between health departments serving different sized populations.

Previous literature has also described differences observed in the number, educational level, and training of staff between health departments serving vastly different sized populations. The NACCHO 2010 National Profile of LHDs described noticeable discrepancies in both the average number of total staff employed and the average number of epidemiologists employed among health departments varying in size of population served (5). The results from this study are consistent with these findings. The small number of epidemiologic staff members observed in small LHDs, while expected, could represent an important deficiency in the structure of these agencies. A health department with as few as the observed average of 2.4 staff members capable and consistently performing epidemiologic tasks could be easily overwhelmed by a large scale public health emergency. Further research is needed to determine the sufficient number of staff necessary to adequately manage the public health needs of a community based on the specific characteristics of both the individual community and health department. For smaller health departments which lack a large number of staff, it is important to assess if existing protocols are in place if these agencies should become extended beyond their capacity. Smaller agencies must have the means to efficiently contact necessary assistance outside their LHD if a situation should arise requiring increased staff and resources. An assessment of this kind was beyond the scope of this study.

The observed strata difference in academic degree distribution is consistent with the widely varying academic degree distribution found in NACCHO’s 2010 National Profile of LHDs when comparing LHDs serving different sized populations (5). The greater advanced degree percentage in large health departments could allow department staffs to more comprehensively manage broad-reaching community health duties, both in day-to-day practice and in the event of a public health emergency, when compared with small health departments. In small departments, the higher percentage of staff holding registered nursing degrees indicates a stronger background in the clinical aspects of public health. These results could also indicate these staff members may perform other duties within their health department outside the realm of epidemiologic functions. As the backgrounds of staff employed at health departments of varying size appear to differ, training needs of staff is also likely to be different. Training of health department staff must be developed based on the background, capability and professional differences of the intended participants, as well as designed to equip attendees with skills and knowledge that they might be previously lacking (27).

A study conducted on the Competencies for Applied Epidemiologists developed by the Centers for Disease Control and Prevention (CDC) and the CSTE found that the education level providing the skills consistent with the lowest tier of epidemiologists was a recent MPH or Masters of Science degree, assuming these professionals had focused on applied epidemiology in their coursework (28, 29). The lack of advanced training in epidemiology observed particularly in small and medium health departments could represent an important deficiency in the ability of these LHDs to adequately fulfill an epidemiologic role within their community.

Higher confidence levels of directors and staff in LHDs serving large populations are consistent with higher levels of performance at larger health departments found in previous studies (21, 22, 30). While expected, these findings could represent an important deficiency in staff capacity and training at LHDs serving smaller and medium populations. However, the variation in lowest reported confidence levels between small and medium strata suggest that size of population served is not the sole variable affecting competency levels of staff. Further research utilizing a larger sample size is needed to more comprehensively explore potential explanations for these observations with sufficient power. Although LHDs serving smaller populations might tend to have higher reliance on outside assistance to adequately fulfill essential epidemiologic duties within a community, all public health workers should have a certain level of proficiency in basic epidemiologic tasks (2).

### Strengths and limitations

The stratified sample for this study was randomly drawn from the NACCHO list of LHDs in an attempt to provide a measure of representativeness of health departments throughout the country. The questionnaire designs were largely based on the questionnaires used by the CSTE in their periodic ECAs and by NACCHO in their National Profile of LHDs. This enabled several comparisons to be made between the results from this study and the results observed in both the ECAs and the National Profiles. When questionnaire responses were obtained via telephone interview, issues of interviewer bias were minimized by reading the questionnaire as written for every participant.

As this was intended as a pilot study, time and budgetary constraints restricted the size of the initial sample chosen. The small sample size, combined with a low response rate, limited the calculation of statistics measuring statistical significance of differences between strata based on a lack of adequate power. While the small sample size limited the ability to conclusively identify the presence of important disparities between LHDs serving different sized populations, observed differences can be used to generate hypotheses for further research on a larger scale. Future study of epidemiologic staff in health departments serving various sized populations can utilize methods from both the design and analysis phase of this study as a model to conduct research using a larger sample to further evaluate the differences observed in results. Bias due to variable collection procedures for questionnaire responses was also possible, as data were collected using both internet questionnaires and telephone interviews. Differences in assumed responsibilities between health departments in different states, jurisdictions, and of different sizes are likely to be present, though a full evaluation of health departments based on inherent differing responsibility characteristics was beyond the scope of this study. Due to the structured nature of the questionnaire, this study was not able to capture any unique characteristics of health departments that rely on outside sources, such as the state health department or an inter-county shared epidemiologist, to enhance their epidemiologic capabilities. The design of this study prevented evaluation of differing opinions in staff competency level by directors and staff within the same department, though this aspect merits further investigation. The challenging nature of designing an appropriate tool for measuring epidemiologic competencies became apparent throughout the course of this study, given the extensive variety of potential staff responsibilities dependent on a health department’s needs and resources. Attempting to define which competency measures were intrinsically useful and which were potentially irrelevant for different health departments was beyond the scope of this study, although this has been explored in previous studies (28, 29). It is also important to note that the variables analyzed within this study included measurements of perceived competency and did not attempt to use validated tools or methods to assess actual task competency levels of staff. Previous evaluations, both within and outside the field of healthcare, have found that individual self-assessment often did not match with actual knowledge and abilities, although self-assessment tools are commonly used in practice (27, 31).

## Conclusion

The purpose of this study was both to provide an initial description of the potential measures of the epidemiologic capacity of LHDs, including the number, education, training, and competency level of department staff, as well as to set groundwork for future assessments of this nature within LHDs. Results of this study can be used as a baseline for the design of larger studies aimed at identifying areas where training and personnel resources might be particularly needed to minimize any deficiencies in the capabilities of LHDs of all sizes. Although it has been noted that every staff member functioning in an epidemiologic role within their health department should not be expected to be equally competent in all areas, the overall epidemiologic staff in each health department should be expected to have a certain combined level of proficiency in epidemiologic tasks (29). Findings from this study suggest that competency levels across a wide variety of categories can be improved in health departments of all sizes. There are several tools available defining competencies in which both public health professionals and epidemiologists in particular, should have a certain level of proficiency (3, 29, 32). Heightened proficiency in outlined competencies can be achieved through enhanced education and ongoing professional training of the public health workforce. Further design and adoption of existing training programs could have a significant effect on improving proficiencies of staff, which could lead to dramatic enhancements in the operation of public health systems.

## Author Contributions

Kaitlin A. O’Keefe contributed to the conception and design of the project, data collection, statistical analysis, interpretation of data, literature review, and manuscript composition. Shira C. Shafir contributed to the conception and design of the project, data collection, interpretation of data, literature review, manuscript review and revision, supervision, and obtaining project funding. Kimberley I. Shoaf contributed to the conception and design of the project, literature review, manuscript review and revision, supervision, administrative/material support, and obtaining project funding.

## Conflict of Interest Statement

The authors declare that the research was conducted in the absence of any commercial or financial relationships that could be construed as a potential conflict of interest.
